# Towards an HIV-free generation in Cuba

**DOI:** 10.2471/BLT.16.021216

**Published:** 2016-12-01

## Abstract

Last year Cuba became the first country in the world to eliminate mother-to-child transmission of HIV and syphilis as public health problems. Other countries are following suit. Andréia Azevedo Soares reports.

Five years ago, Yusleidys Fernandez looked in the mirror and saw rashes on her face. She went to a community-based polyclinic in the Cuban capital, Havana, for a check-up.

A physician looked at her skin lesions and offered her an HIV test. The initial result was positive. Follow-up testing confirmed the distressing news.

“I was so scared. I couldn’t believe it was happening to me. I felt devastated. I was so young and had not even had children,” says Fernandez.

Now at 29 years, Fernandez is the proud mother of a healthy three-year-old girl called Lola. Lola is one of 112 healthy babies born to mothers with HIV in Cuba in 2013. That year, only two babies were born with HIV in the Caribbean country and only three babies were born with congenital syphilis.

**Figure:**
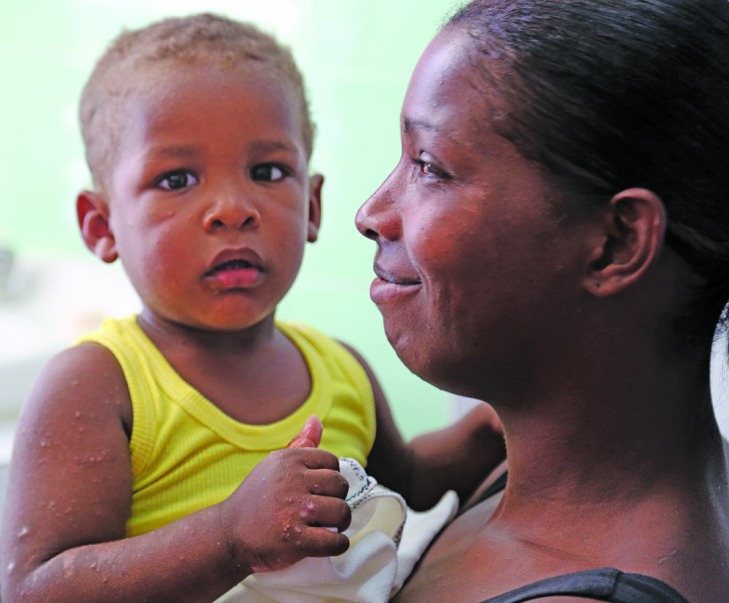
Mother living with HIV holds her child, who is not HIV positive, at the Policlínico Bernardo Posse in Havana, Cuba

WHO/PAHO/S Oliel

More than a million pregnant women are infected with syphilis worldwide, a debilitating chronic bacterial disease that can be fatal for their babies but that is treatable with penicillin. The World Health Organization (WHO) and its partners are working with countries to prevent the transmission of both HIV and syphilis from mother to child.

On 30 June 2015, Cuba became the first country in the world to receive official recognition from WHO for having eliminated mother-to-child transmission of HIV and syphilis as public health problems.

Cuba’s success was followed by similar news from Belarus and Thailand. Armenia has since received WHO recognition for having eliminated mother-to-child transmission of HIV and Moldova for having eliminated congenital syphilis.

Elimination is defined as fewer than 50 babies born with HIV infection per 100 000 live births. In addition, mother-to-child transmission rates of HIV should be less than 2% a year.

The HIV epidemic in Latin America and the Caribbean countries began in the 1980s. Cuba was one of the first countries in the region to rollout an HIV prevention and treatment programme, which now provides antiretroviral therapy to all people with HIV.

As a result of programmes like this in the region, the prevalence of HIV infection among pregnant women in these countries is about 0.5% or less.

Since 2010, experts from WHO headquarters and the Pan American Health Organization (PAHO), WHO’s Regional Office for the Americas, have been working with Cuba and other Latin American and Caribbean countries to implement a regional initiative; the goal is to reduce mother-to-child transmission of HIV to a very low level “such that it is no longer a public health problem” according to WHO’s *Global guidance on criteria and processes for validation: elimination of mother-to-child transmission of HIV and syphilis* published in 2014.

In 2013, nine countries and territories in the Americas reported data suggesting that they had achieved or were close to these elimination goals. Cuba was one of them. (The others were Anguila, Barbados, Canada, Jamaica, Montserrat, Puerto Rico, Saint Kitts and Nevis, and the United States of America.)

In fact, Cuba had been reporting such data three years earlier according to Dr María Isela Lantero, head of Cuba’s national HIV/AIDS programme.

“In the case of congenital syphilis, we already had indicators showing that we had eliminated the problem since the 1980s,” says Lantero. 

“With HIV it took longer. In the beginning of the epidemic in our country few women got pregnant. It was normal: there was a lot of fear. We didn’t know much about the disease and we didn't have the disease control methods we have today,” Lantero says.

Pregnant women with HIV who are not receiving antiretroviral therapy have a 15–45% chance of transmitting the infection to their babies during pregnancy, labour, delivery or breastfeeding. If they are on antiretroviral medicines, that risk drops to about 1%. There is no cure for HIV infection, but antiretroviral therapy can control the virus and reduce the risk of transmission.

In Cuba, pregnant women (and their partners) are tested for HIV and syphilis every trimester; those found to be HIV positive are given antiretroviral drugs and caesarean deliveries and can buy heavily subsidized milk formula.

Such strategies have allowed the majority of pregnant women living with HIV in Cuba to lead a productive life and have healthy babies, like Lola.

For medical anthropologist Arachu Castro, Cuba’s success is largely thanks to its strong primary health-care network. “This network is the backbone of the Cuban health-care system and it works at community level.

“So it’s easy to access prenatal care because there is a family physician-and-nurse clinic in every neighbourhood,” says Castro, the Samuel Z Stone Chair of Public Health in Latin America at Tulane University in New Orleans in the United States of America.

Cuba’s 451 community-based polyclinics are considered the main pillar of the island’s health-care system and address most of the health conditions of the population.

In 2015, 92% of the 21 250 people with HIV in Cuba were receiving care and counselling at polyclinics and family physician-and-nurse clinics. In addition, Cuba has a network of more than 230 laboratories that test people for HIV, syphilis and other infectious diseases.

“In Cuba, physicians and nurses are part of the community, they know their patients by name.”

Arachu Castro

“As a whole, it’s a health system that really responds to the needs of the population,” Castro says. “In Cuba, physicians and nurses are part of the community, they know their patients by name and they are familiar with the social pressures or local problems, such as lack of electricity or water.”

Cuba's focus on health promotion and primary care has paid off. The prevalence of HIV on the Caribbean island has always been the lowest in the Americas and among the lowest in the world, according to the Joint United Nations Programme on HIV/AIDS.

In 2014, HIV prevalence among adults in Cuba aged between 15 and 49 years was about 0.2–0.3%. The country started providing prophylaxis to prevent mother-to-child transmission of HIV early on and in the early 1990s physicians were prescribing zidovudine to pregnant women with HIV.

In 2001, Cuba developed several generic medicines for HIV infection. Thus antiretroviral therapy became the standard treatment and has always been free of charge for patients.

Cuba produces five generic medicines for HIV and these are combined with other HIV medicines that are procured outside the country with funding from the Global Fund to Fight AIDS, Tuberculosis and Malaria. Today antiretroviral therapy is prescribed free of charge to all pregnant women and babies with HIV infection, regardless of their clinical or immunological status.

In addition, health workers explain the benefits of having a caesarian section to reduce the risk of transmission compared with a vaginal birth, and the importance of using condoms during and after the pregnancy. 

**Figure:**
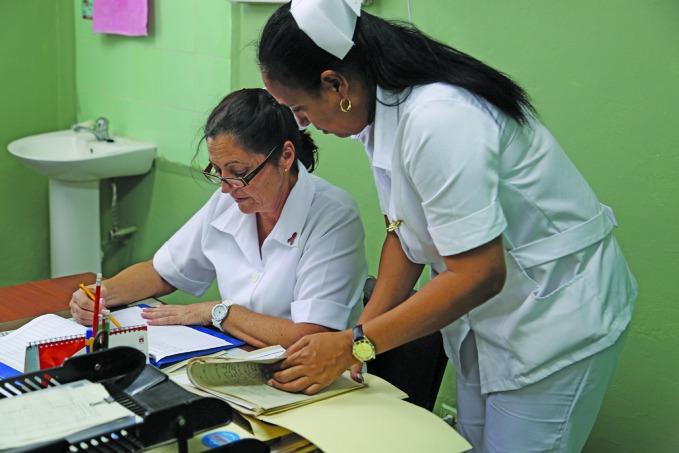
Health workers at the Policlínico Bernardo Posse in Havana, one of Cuba’s 451 community polyclinics

WHO/PAHO/S Oliel

In contrast to WHO guidelines that advise women with HIV in low- and middle-income countries to breastfeed their babies while they are on antiretroviral therapy, such women in Cuba are strongly recommended to feed their babies with formula milk that is suitable for newborns. Additional food rations are provided to these patients so that they can buy this.

“We are taking one step at a time. Currently Cuba is not encouraging women with HIV to breastfeed their babies. We have confidence in our replacement feeding, which is safe and affordable for these mothers,” says Lantero.

In November 2013, Cuba applied to WHO to validate its elimination of mother-to-child transmission of HIV and syphilis.

To support this, Cuban health officials prepared a country report in 2014, which was reviewed by independent experts.

Apart from reducing HIV transmission from mothers to their babies to a very low level, countries are required to demonstrate their political commitment to elimination targets, strong maternal and child health and disease control programmes, reliable laboratory services and a robust health information system. Respect for human rights and compliance with the principles of gender equality and civil society engagement are also essential.

An in-country assessment was carried out by international experts the following year. Their recommendation to validate Cuba’s application was endorsed by a committee of 17 external experts that advises WHO headquarters on the validation of the elimination of mother-to-child transmission of HIV and congenital syphilis.

On 30 June 2015, WHO announced that Cuba had made this major step towards an AIDS-free generation.

The validation process also recognizes the fact that Cuba achieved this while protecting the rights of women with HIV, which means that all health services must be provided free of coercion. This was not always the case in Cuba.

Between 1986 and 1993, Cubans found to be HIV-positive were forced to live in sanatoria for people with acquired immune deficiency syndrome (AIDS). These facilities are now called Centres for Comprehensive Care for People with HIV/AIDS and mainly provide ambulatory HIV care and education on how to live with the virus. This education includes information on the importance of healthy diet, good personal hygiene and compliance with treatment.

World AIDS Day is an opportunity to show support to the 36.7 million people living with HIV worldwide. This year, the WHO campaign focuses on different aspects of HIV prevention including reduction of mother-to-child transmission.

“This is what I have to say on World AIDS Day: being infected does not stop you from having a family and having HIV is not the end of the world. If you take the medication and work with your physicians to achieve a minimal viral load, you can have a wonderful baby,” says Fernandez.

“Being infected does not stop you from having a family and having HIV is not the end of the world.”

Yusleidys Fernandez

